# Evaluation of Machine Vision-Based Automated Detection of Freeway Incidents

**DOI:** 10.3390/s26206451

**Published:** 2026-10-12

**Authors:** Chonlachart Jeenprasom, Tanchanok Sirikanchittavon, Mohammed Hadi, Zhenyu Wang

**Affiliations:** 1 Department of Civil and Environmental Engineering, Florida International University, 10555 West Flagler Street, EC 3730, Miami, FL 33174, USA; cjeen001@fiu.edu (C.J.); tsiri001@fiu.edu (T.S.); 2 Center for Urban Transportation Research, University of South Florida, 4202 E Flowe Ave, CUT100, Tampa, FL 33620, USA; zwang9@usf.edu

**Keywords:** automated incident detection, computer vision, freeway incidents

## Abstract

Automatic incident detection (AID) can significantly decrease the time to detect incidents and better identify incident locations to allow faster response. The deployment of traffic surveillance cameras has motivated transportation agencies to investigate computer vision technologies for AID using live video streams from these cameras. This paper evaluated three commercially available computer vision-based AID systems. The evaluated AID systems were able to use video streams from six existing Pan–Tilt–Zoom (PTZ) surveillance cameras for incident detection when these cameras were on predefined presets. The three systems with the selected settings performed differently. For example, the system with the highest number of true stopped vehicle incident detections also had a relatively high number of false alerts. The AID system accuracy also varied by study site, likely due to traffic patterns, roadway geometry, and camera angles. Two major factors contributed to false alerts: stop-and-go traffic during non-incident conditions and other highway objects, including light reflection and glare, that were falsely detected as incidents. The three systems also detected additional incidents not reported in existing incident management databases, but these were mainly shoulder incidents and work zone vehicles.

## 1. Introduction

A critical task for transportation agencies is incident management, including detecting, responding to, and clearing traffic incidents. An effective incident management system is expected to restore traffic flow as safely and quickly as possible and improve the safety of road users and crash victims [1]. The adverse impacts of traffic incidents (e.g., congestion, delays, and secondary crashes) depend on two key characteristics: the reduction in facility capacity and the duration of the incident and lane blockages [2]. Automatic incident detection (AID) can significantly decrease the time to detect incidents and to better identify incident locations to allow faster response. The developments and implementations of AID have focused for many years on using traffic sensor data (volume, speed, and occupancy) [3]. Several AID algorithms have been proposed and used to detect incidents based on sensor data. These algorithms use comparative analysis, traffic modeling, statistical analysis, and machine learning/artificial intelligence approaches.

Closed-Circuit television (CCTV) cameras have long been used by traffic management center (TMC) operators for incident verification. CCTV cameras are typically installed along roadways with typical spacings of about one mile on urban freeways. These cameras are equipped with pan–tilt–zoom (PTZ) functionality, allowing TMC operators to monitor real-time traffic conditions over a broad field of view.

Transportation agencies have used CCTV cameras mainly for the verification of incidents and for gathering additional information about these incidents after the incidents are detected by other manual or automated means. The availability of the CCTV cameras and the associated infrastructure has motivated transportation agencies to investigate solutions that utilize video analytics of the camera video feeds received at the TMC to detect and better locate incidents. The potential of using video analytics in combination with the already available video surveillance infrastructure can be very powerful and cost-effective, if the performance of the video analytics solution can be verified.

Video analytics products are becoming available from commercial vendors. These vendors report that their products can process CCTV surveillance camera feeds automatically through computer vision technologies in real-time and apply incident detection algorithms to identify abnormal traffic conditions. However, these vendors admit that better incident detection results are expected with the use of fixed cameras installed specifically for the purpose of incident detection based on CCTV camera video feeds. This is particularly because the TMC operators usually change the field of view of these cameras as needed in their traffic monitoring and incident verification processes. The effort summarized in this paper evaluated three commercially available computer vision-based AID systems for incident detection based on CCTV camera video feeds.

## 2. Review of Computer Vision-Based AID

The core components of computer vision-based incident detection systems include the Video Image Processing (VIP) module, which extracts features from video footage, and the incident detection algorithm, which analyzes these features to identify abnormal traffic conditions [4].

### 2.1. Video Image Processing

Kastrinaki et al. [5] conducted a thorough review, description, and classification of methods and technologies of VIP used in traffic applications. VIP for traffic monitoring normally involves two major tasks of perception: (a) recognition of the road geometry that constitutes the image background, and (b) detection or tracking of vehicles in the image. This allows the acquisition and analysis of traffic measures, such as presence and numbers of vehicles, speed measurements, queue lengths, occupancy, and so on. A VIP system detects vehicles by analyzing changes between successive video frames, examining the variations across pixel groups to differentiate moving objects from the background [6]. To improve accuracy, the algorithms filter out visual disturbances caused by weather, shadows, and lighting transitions from day to night.

VIP systems can be categorized into three types based on the method used to detect and monitor vehicles within the camera’s field of view: tripline, closed-loop tracking, and data association tracking methods [4]. The tripline systems function similarly to point traffic detectors like inductive loops or microwave detectors by defining fixed detection zones where changes to pixels are used to signal vehicle presence. Closed-loop tracking systems expand the monitoring area by continuously tracking vehicles across the field of view, confirming detections through multiple observations. Data association tracking systems represent a more advanced framework that identifies and tracks individual vehicles by matching moving objects across frames, allowing for continuous and accurate tracking over time. Coifman et al. [7] identified and described four VIP vehicle tracking methods.

Recent advances in computer vision have increasingly shifted traffic video analysis from traditional image-processing methods toward deep learning-based object detection and tracking. Modern approaches use convolutional neural networks and related architectures to identify and track vehicles, pedestrians, and other roadway objects directly from video streams. Recent research on traffic-video object detection has highlighted the improved capability of deep-learning methods while also identifying challenges associated with illumination, occlusion, camera viewpoint, image quality, and changing traffic environments [8]. Recent studies have also demonstrated the application of these deep-learning methods to traffic surveillance video. Shokri et al. [9] developed a vehicle detection and tracking framework based on the Segment Anything Model (SAM) using videos from uncalibrated, low-resolution traffic surveillance cameras. Nocua et al. [10] evaluated several convolutional neural network architectures, including MobileNet-SSD, YOLOv5, and Faster R-CNN, and combined object detection with Lucas–Kanade optical-flow tracking for real-time traffic monitoring.

### 2.2. Incident Detection Algorithms

The extracted features using VIP are analyzed to determine whether an incident has occurred using an incident detection algorithm. Traditional AID methods include comparative algorithms, statistical methods, time-series models, traffic-flow models, and early artificial intelligence approaches [11]. More recent research has increasingly applied machine learning and deep learning to distinguish normal and abnormal traffic states and improve the adaptability of incident detection systems.

Pan et al. [12] demonstrated the use of a hybrid traffic-flow and deep-learning framework for traffic-state estimation under congested conditions, illustrating the growing application of data-driven methods for identifying changing traffic conditions relevant to AID. ElSahly and Abdelfatah [13] developed a machine-learning-based freeway incident detection system using an artificial neural network. The model was designed to distinguish normal and incident traffic patterns while considering congestion level, incident severity, incident location, and detector spacing. The study reported a detection rate of 95.6%, a false alarm rate of 1.01%, and a mean time to detection of 0.89 min during training, demonstrating the potential of machine-learning approaches for freeway AID.

In parallel, advances in computer vision and deep learning have enabled incident detection to move beyond traffic-parameter-based methods toward direct analysis of roadway video. These approaches use object detection, tracking, trajectory analysis, and video anomaly detection to identify stopped vehicles, pedestrians, crashes, and other abnormal events. Khan et al. [14] applied a convolutional neural network-based approach to classify traffic incidents as anomalous events in surveillance videos. Pawar and Attar [15] formulated traffic incident detection as a one-class anomaly detection problem and used spatiotemporal and sequence-to-sequence autoencoders to learn normal traffic patterns and identify deviations associated with incidents. More recently, Bartolomé-Hornillos et al. [16] developed a self-adaptive video-based AID framework that combines deep-learning-based object detection and tracking with rule-based incident detection to identify abnormal vehicle speeds, pedestrians or objects on the roadway, stopped vehicles, and wrong-way movements. Meng et al. [17] further proposed a multimodal framework that combines visual video features with textual information to perform fine-grained detection and classification of different highway traffic anomalies.

### 2.3. Evaluation of Computer Vision-Based AID

Previous studies have evaluated the performance of computer vision technologies for traffic detection under various environmental and operational conditions. Wan et al. [18] developed a system for detecting stopped vehicles and collecting traffic data such as speed and volume. Field experiments demonstrated speed accuracy of 91% and volume accuracy between 90–94%. Grant et al. [19] assessed the use of computer vision for planning applications and found that the accuracy was sensitive to detection distance, lighting glare, weather, and camera vibration. They recommended quality control procedures and optimal detector placement to ensure reliable data.

The performance of AID systems based on machine vision is expected to vary depending on whether the video data are collected using fixed cameras or pan–tilt–zoom (PTZ) cameras for automated video analysis. Dedicated fixed cameras maintain a consistent field of view, orientation, zoom level, and image geometry after installation and calibration, allowing detection zones and lane boundaries to remain stable. In contrast, the views of PTZ cameras are expected to be repositioned during traffic management operations, resulting in changes in viewing angle, object scale, roadway geometry, and lane position within the image. Recent studies have identified changing or moving camera views as an additional challenge for video-based anomaly detection [20]. PTZ camera views may further deviate from their original presets over time because of camera replacement, maintenance activities, mechanical slippage, bracket movement, or impacts to camera support structures [21]. Such deviations can affect the calibrated field of view and potentially reduce automated detection performance.

Previous field evaluations provide useful frameworks for assessing video-based AID using existing PTZ camera infrastructure. Across these studies, system-generated alerts were generally evaluated through manual verification using still images, recorded video, and available agency incident records, allowing alerts to be compared against independently observed traffic events and common performance measures included detection rate, false or noncritical alarm rate, and mean time to detect [22,23]. In addition to these alert-based measures, some evaluations also considered the influence of camera configuration and view stability by comparing false alerts when cameras were at versus away from their predefined home views [24]. These evaluations indicate that PTZ-based AID performance can be assessed using verified detections, false alerts, and detection timeliness, while also considering whether the camera remains at its predefined view.

Other studies have examined how camera and environmental conditions affect these performance measures. Prevedouros et al. [4] tested three video detection systems in freeway tunnels using staged incidents. Detection rates varied widely from 21% to 71%, and the false alerts were found to be influenced by lighting effects, tunnel curvature, and camera angle. The study concluded that system configuration and calibration were crucial for reliable detection. Martin [25] found an average incident detection accuracy based on video of 87% in daytime and dusk, 73% at night, and 81% in snow. False or missed detections occurred in 17% of the cases, often due to glare, snow, vibration, or occlusion. The study emphasized proper camera angles, stability, and maintenance to improve reliability. Kim et al. [26] conducted a study on how video quality impacts detection rates. Using three video settings (low to high), the detection improved from 25.0% to 80.4%, and the false alerts dropped from 27.7% to 12.5%. Weather and lighting significantly affected performance. Camera height had mixed effects, while preset deviation consistently reduced accuracy. Recent traffic-video research has also highlighted the effects of camera viewpoint, illumination, image quality, and occlusion on computer vision performance [8].

Collectively, these evaluations underscore the potential of video analytics for incident detection, while also highlighting the critical importance of video quality, camera setup, lighting, weather, and calibration in ensuring reliable and accurate system performance. Fixed cameras provide a more stable environment for automated detection, whereas PTZ cameras introduce additional challenges because their field of view can change during normal TMC operations. However, PTZ cameras offer the potential to use existing agency surveillance infrastructure without installing additional dedicated fixed cameras. Limited research has compared multiple commercially available AID systems using the same existing operational PTZ camera infrastructure under a common evaluation framework. This study addresses this gap by evaluating three commercial systems using the same PTZ camera feeds, incident definitions, verification procedures, and performance measures.

## 3. Deployed Systems and Evaluation Method

This section presents a review of the deployment of the three AID systems evaluated in this study and the method used in their evaluation.

### 3.1. AID System Deployment

Three commercial AID systems were selected for the evaluation in this study. The systems utilize object tracking computer vision technologies for event detection. They continuously monitor video streams from the CCTV cameras and send alerts via email if an incident is detected. Incident log data, including video clips or snapshots, are recorded in this study for evaluation purposes. The three systems—referred to in the rest of this paper as System 1, System 2, and System 3—process live video streams (1920 × 1080 pixels at 30 frames per second) from cameras to identify incidents using computer vision technologies. The study leverages video streams from six existing PTZ cameras of the Florida Department of Transportation (FDOT) District 4 in Broward County, FL. The AID systems require the cameras to be fixed at one position with a constant view angle and zoom setting to effectively detect the incidents. The CCTV cameras were set at a fixed view (Preset) unless TMC operators moved them to another position to confirm and validate incidents. If there is no camera operation within 15 min after moving a camera from its preset, the camera will return to its Preset. The AID systems were installed and run on local computers located in FDOT District 4 TMC to meet the FDOT requirements and operating procedures.

**Figure 1:**
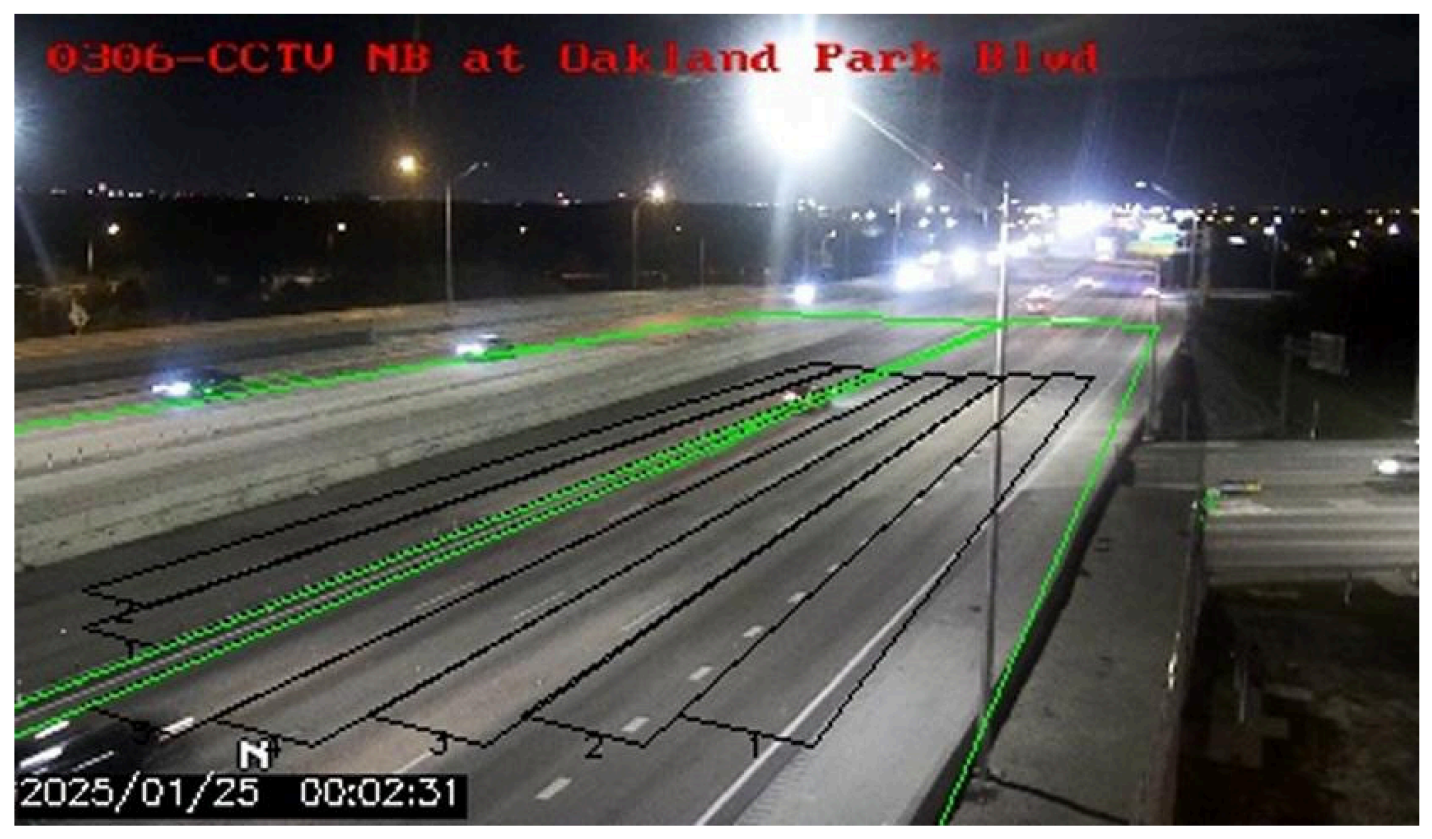
An example of detection zones for one of the systems.

The vendors were responsible for calibrating their systems to achieve the best possible performance under the conditions at each study site. The calibration process included three primary components:

- Geometry Configuration—The vendors defined detection zones, which represent the spatial limits for incident detection, for each CCTV camera. To distinguish the lateral position of detected incidents, vendors also labelled travel lanes in their model calibration. An example of a detection zone (green polygons) and an example of lane delineation (black polygons) are shown in Figure 1. The three AID systems had specific detection zone definitions and lane delineations that the vendors selected to optimize the performance of their systems. In general, the AID systems had a limited detection range, typically between 200 and 700 feet within the camera preset’s field of view. The lateral coverage of the AID system is not uniform across sites and vendors. At some locations, the detection zones selected by the vendor encompass both travel directions, while at others, the coverage is limited to a single side of the roadway.
- Model Calibration—The three vendors calibrated the parameters of their computer vision models to ensure that the models performed the best in detecting and tracking vehicles, pedestrians, and other objects within the detection area.
- Verification of System Performance—The AID systems identify incidents by monitoring whether a vehicle remains stationary beyond a threshold and trigger incident alerts for stopped vehicles upon such identification. These thresholds were set by vendors and were varied across different locations (shoulder or travel lane) and traffic patterns (congestion or free flow).

To provide a consistent evaluation framework, all three systems were evaluated using the same existing FDOT PTZ camera infrastructure and were assessed using the same incident definitions, verification procedures, and performance measures. Table 1 summarizes the available technical and operational characteristics of the three AID systems in this study.

### 3.2. Deployed System Exploration

The research team conducted an initial exploration of the three systems to understand and verify their functions. This exploration included:

- Verifying the functions and capabilities of each system.
- Understanding the user interfaces and parameter settings of the systems.
- Checking and verifying the existence of detection log data.
- Verifying the incident detections and the logged videos associated with detected incidents.

Each AID alert was manually reviewed using the associated video clip recorded by the corresponding system. Two members of the research team participated in the verification process. Each alert was first checked to determine whether the camera was positioned at its predefined preset at the time of detection. Alerts generated while the camera was on preset were then classified as either a True Alert or a False Alert based on the visual evidence available in the recorded video.

- A True Alert was defined as an alert for which the reported event type was visually confirmed within the system’s detection zone at the time of detection. For example, a stopped-vehicle alert was considered a True Alert when a vehicle was observed to be stationary within the detection zone not because of congested traffic conditions, while a pedestrian alert was considered a True Alert when pedestrian presence was visually confirmed.
- A False Alert was defined as an alert for which the reported event type could not be visually confirmed within the detection zone. Examples included vehicles temporarily stopped during stop-and-go traffic or other objects, such as shadows, light reflections, glare, traffic signs, or construction devices, that were incorrectly identified by the system.

Two of the three AID systems were tested from 14 January to 28 February 2025. The third system was tested from 14 January to 14 March since it had a period during which it was not functioning correctly, as explained later. The analysis focused on the ability of the systems to detect stopped vehicles and pedestrian presence in the peak period (6:30 am to 9:30 am and 4:00 pm to 7:00 PM) and the off-peak period. In addition to doing the analysis for different congestion levels (peak vs. off-peak periods), the analysis was done under different lighting conditions (daylight vs. nightlight) and weather conditions (dry versus rain). The detections were filtered to retain only those captured while the CCTV was on the predefined presets. During the test period, the research team collected detection event data generated by the systems including the identification of the source camera, detection timestamp, detection type (stopped vehicle, pedestrian presence, or congestion events), and video clips to verify the events.

The research team also retrieved FDOT incident records from the Regional Integrated Transportation Information System (RITIS) that acts as the data warehouse for the FDOT traffic and incident operations and management systems. The performance measures used in the evaluation include:

- False Alert Rate (FAR): This is the proportion of false alerts compared to the total number of alerts generated by each vendor. A false alert is defined as an incident detection that could not be verified based on the video footage.

(1) $$\mathrm{FAR}=\frac{\mathrm{False alerts}}{\mathrm{Total Alerts}}\times 100\%$$

On the other hand, precision measures the proportion of correct positive predictions (true positives) out of all cases the model predicted as positive.

(2) $$\mathrm{Precision}=\frac{\mathrm{True Alerts}}{\mathrm{Total Alerts}}\times 100\%$$

Precision is directly related to FAR based on their definition:

Precision = 1 − FAR(3)

Since FAR is the complement of precision, this study adopted FAR in the analysis. A high FAR indicates poor precision.

In addition, a complete ground-truth record of all incidents occurring within the camera detection zones was not available. Identifying false-negative events would have required continuous video recordings throughout the study period so that all actual incidents could be identified and compared with the alerts generated by each system. Because continuous recordings were unavailable (the recording was only available for the conditions identified by the systems as incident conditions), the number of incidents that occurred but were not detected could not be determined. Therefore, recall could not be calculated.

- Matching Rate: This is the number of detected incidents that could be matched with the incidents archived in the FDOT database. The matching rate was evaluated as the number of alerts that matched RITIS-recorded incidents within a 0.5-mile radius of the camera location and ±30 min from the time the incident was detected by the evaluated AID. These spatial and temporal thresholds were selected to account for differences between the actual location and timestamp of an incident detected by the AID systems and those recorded in the FDOT database. However, to assess the sensitivity of the matching results to these thresholds, an analysis was conducted using alternative spatial and temporal thresholds. The spatial threshold ranged from 0.25 to 1.00 miles, and the temporal threshold ranged from ±15 to ±60 min. This sensitivity analysis was conducted using all true stopped-vehicle alerts. The incidents archived in the FDOT database are detected by two sources. The two sources are “Waze Provided”, which is based on a real-time data stream received by FDOT from a third-party vendor (Waze), and “FDOT Recorded,” which is recorded by TMC operators based on information from highway patrol, service patrol and other sources. This study matched the AID-detected data with the data of both sources. The matching rate is calculated as follows:

(4) $$\mathrm{Matching Rate}=\frac{\mathrm{Alerts that matched with FDOT archived data}}{\mathrm{Total Alerts}}\times 100$$

- Latency of Detection: This is the time difference between the timestamp of a matched AID-detected incident and the corresponding time stamp in the existing FDOT database.
- Hypothesis Testing for Detection Timeliness: A statistical analysis was conducted to determine whether the AID system detection time was significantly earlier than the FDOT recorded time for the matched incidents. The null hypothesis is that the AID is not earlier than the reported incident start time in the FDOT archived database. A one-tailed permutation test, which is a non-parametric test, was used to assess the median difference in the detection times.

In addition, this study developed a binary logit model to identify the factors contributing to false alerts in stopped vehicle detections. The features investigated for inclusion in the model were the specific investigated system, the camera providing video feed for detection, travel lane vs. shoulder detection, rain versus dry conditions, peak vs. off-peak hours, and nightlight vs. daylight conditions.

## 4. Results

This section discusses the results obtained from the evaluation of the computer vision-based AID systems in this study. The discussion addresses the number of detected incidents, false alert rates, causes of these false alerts, matching of the incidents detected by the AID systems with the recorded incidents in the FDOT database, and latency analysis that estimates the difference between the AID system detection and the timestamps of the incident start time in the FDOT database.

### 4.1. Number of Detections

Table 2 indicates that, when considering both true and false detections, System 2 detects the highest number of stopped vehicle incidents, both in the total number of incidents and the incidents detected while the cameras are on presets. The number of pedestrian detections per day for System 2 and System 3 was close and higher than what was detected by System 1. There were a large number of incidents detected when the cameras were not on preset, particularly in the case of System 2. These incidents were not considered in this study since a large proportion of them were either false alerts or alerts issued when the operators moved the camera to zoom in on incident sites after the incidents were detected by other means.

### 4.2. False Alerts

**Figure 2:**
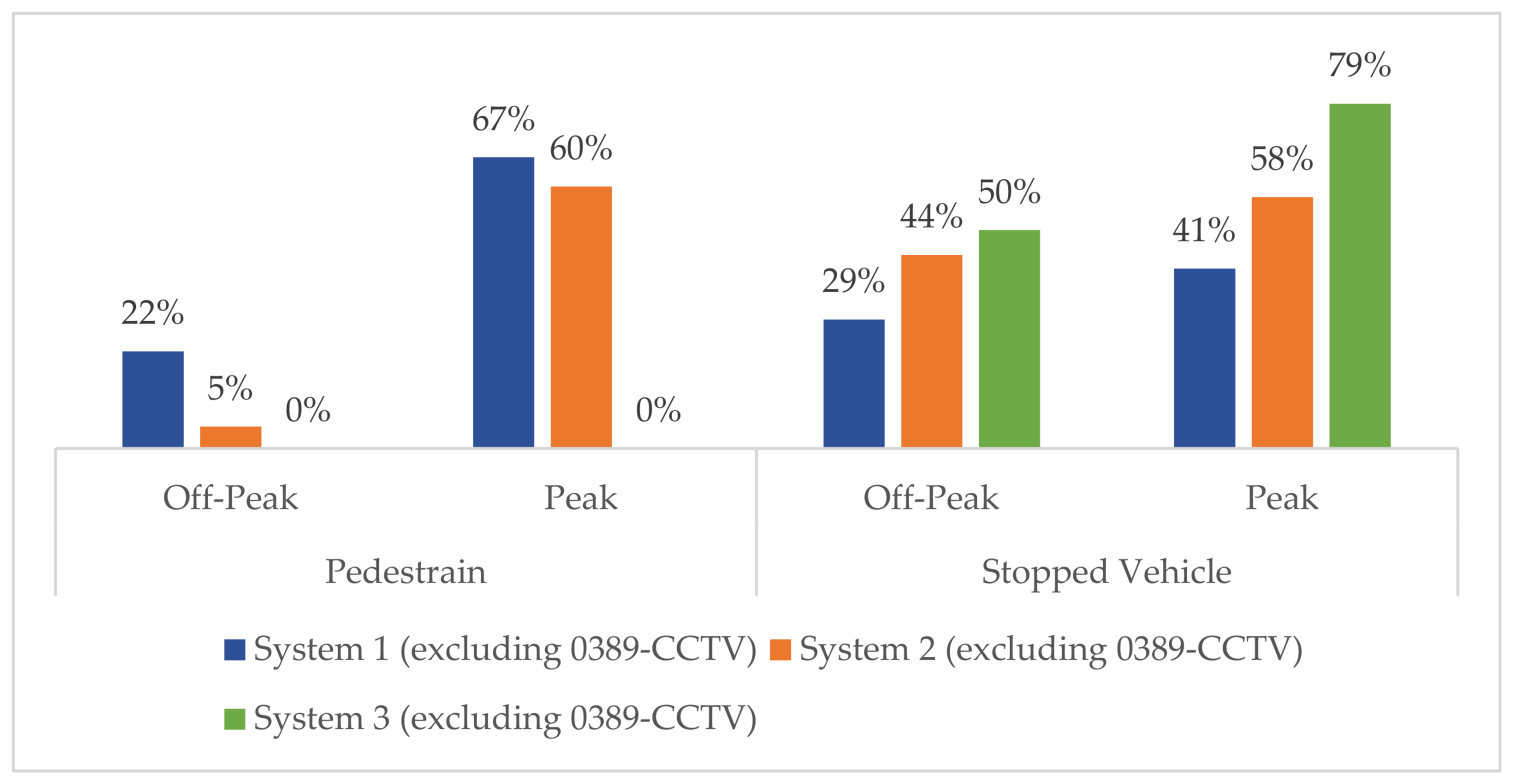
False Alert Rates (FARs) by system, time period, and incident type.

The FAR analysis revealed notable differences in detection performance across different systems. The examination of the results indicates that the detection of one of FDOT (Broward County, FL, USA) cameras (Camera 0389-CCTV) contributed to a large proportion of the false alerts of the AID systems due to light reflection and glare at night. To provide a consistent comparison across systems, the FAR values are reported in Figure 2 without detection using that camera’s video. For the stopped vehicle incidents during the off-peak period, System 1 had the lowest FAR (29%), followed by System 2 (44%), and then System 3 (50%). During the peak periods, System 1 again had the lowest FAR of 44%, followed by System 2 (58%), and then System 3 (79%).

For pedestrian incident detection during the off-peak period, System 3 had a FAR of 0%, while the FARs for System 1 and System 2 were 22% and 5%, respectively. During the peak periods, System 3 maintained a 0% FAR. However, the FARs for System 1 and System 2 were 67% and 60%, respectively, again showing significantly higher FARs in the peak periods compared to the off-peak periods.

The research team examined the attributes associated with the true and false alerts generated by the three systems. It found that the major contributors to the false alerts were the stop-and-go operations and the presence of other objects, as described below. During the peak period, due to the stop-and-go operations, the systems may identify a stopped vehicle due to congestion as an incident. In this regard, there is a trade-off in setting the thresholds of the minimum vehicle stop time that is considered as an incident. Lower time thresholds cause higher numbers of detections but higher numbers of false alerts. As stated earlier, the system vendors were given the freedom of setting their systems’ thresholds. In addition to the stop-and-go operations in the congested period, another major contributor to the FAR is the identification of the presence of other objects as stopped vehicles or pedestrians including objects like shadows, roadside features, vehicle light reflection and glare, traffic signs, and/or construction barrels. These false detections can be reduced by enhancing the object classification algorithms of the utilized computer vision technologies and refining the detection zone configurations.

All false alerts of pedestrian presence generated by System 1 and System 2 were caused by the presence of other objects. System 3 did not generate any false alerts for the pedestrians.

For stopped vehicle detection, Table 3 indicates that the main cause of the false alerts for System 1 was the stop-and-go operations in both the off-peak and peak periods. The false alerts were much higher for System 2 compared to System 1. In the off-peak, the presence of other objects was the main contributor to the false alerts for Systems 2 and 3. In the peak periods, the contribution of the stop-and-go operations to false detection was higher than in the off-peak for Systems 2 and 3. The number of false detections due to fixed objects is much lower in the peak periods compared to the off-peak period for these two systems. Further examination indicates that this is because the off-peak period includes night conditions with light reflections and glares that were falsely identified as incidents.

### 4.3. Composition of True Alerts

**Figure 3:**
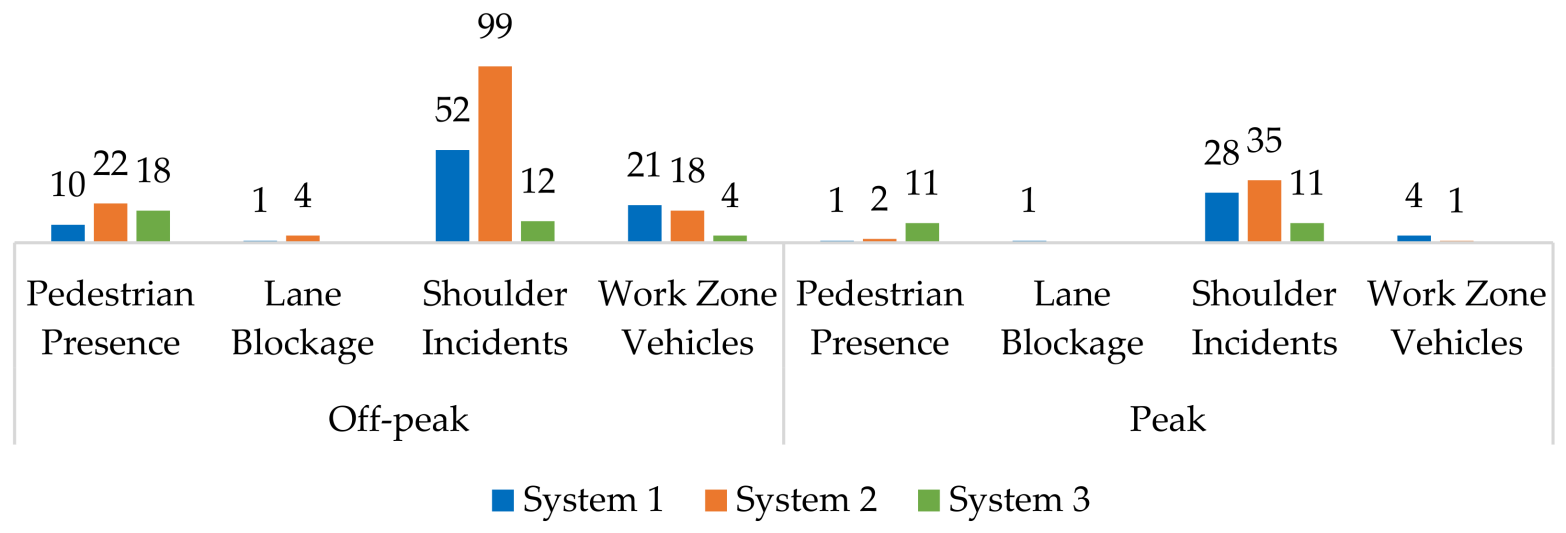
Verification of the composition of “true” detections.

System 2 had the highest number of detections of true incidents followed by System 1, as shown in Figure 3. Figure 3 summarizes the composition of the true detected alerts for the three systems. Large proportions of the detected incidents were shoulder incidents, followed by work zone activities, followed by pedestrian presence detection. Only a few lane blockage incidents were detected by the systems. This could be in part because of the short length of the detection zone that ranges from 200 ft to 700 ft, depending on the system and camera locations. Since lane blockage events have much lower frequencies than shoulder incidents, the expected number of lane blockage events in the detection zone is low.

### 4.4. Impact of Lighting and Weather Conditions

The research team examined the impact of lighting and weather conditions on the alert frequency and FAR. The AID systems operated continuously, 24 h per day, during their respective valid study periods. For the lighting analysis, daytime was defined as 7:00 AM to 6:00 PM, while the remaining hours were classified as nighttime. Systems 1 and 2 had a total of 506 daytime hours and 598 nighttime hours during the 46-day study period. System 3 had 473 daytime hours and 559 nighttime hours during its 43 valid observation days.

Because Camera 0389-CCTV was excluded from this analysis, five cameras were included for each system. The daytime and nighttime exposure durations were therefore 2530 and 2990 camera-hours, respectively, for Systems 1 and 2. For System 3, the corresponding exposure durations were 2365 daytime camera-hours and 2795 nighttime camera-hours.

Table 4 shows that the number of pedestrian detections at night was lower for all systems. System 1 produced eight pedestrian alerts during the day and four at night, System 2 produced 20 during the day and one at night, and System 3 produced 21 during the day and five at night. These differences may reflect lower nighttime pedestrian activity rather than differences in system performance.

Fewer stopped vehicle alerts were also recorded at night. System 1 produced 75 daytime alerts and 37 nighttime alerts, while System 2 produced 126 daytime alerts and 116 nighttime alerts. System 3 exhibited the largest difference, with 50 daytime alerts and 10 nighttime alerts.

Weather conditions were classified as rainy or dry using rainfall observations obtained from the Automated Surface Observing System (ASOS) station at Fort Lauderdale–Hollywood International Airport. Systems 1 and 2 experienced approximately 55.27 h of rain and 1048.73 h of dry conditions. For System 3, after excluding periods during which the system was unavailable, the corresponding exposure durations were approximately 46.27 h of rain and 985.73 h of dry conditions.

After accounting for the five cameras included in the analysis, these durations corresponded to approximately 276.35 rainy camera-hours and 5243.65 dry camera-hours for Systems 1 and 2. For System 3, the corresponding exposure durations were approximately 231.35 rainy camera-hours and 4928.65 dry camera-hours.

Table 4 indicates that the FAR for System 1 for pedestrian presence was 25% during the day and 50% at night. Systems 2 and 3 recorded no false pedestrian alerts at night, but each system had five or fewer nighttime pedestrian alerts. For stopped vehicle detection, the FARs of Systems 1 and 3 decreased, while the FAR for system 2 increased significantly at night because of light reflection and glare.

The research team also compared FAR under clear and rainy conditions. No pedestrian detections were recorded during rainy conditions. For stopped vehicles, System 3 provided no alerts and System 1 provided only one alert that was false. The small number of detections could be due at least in part to the shorter monitored periods with rainy conditions. However, System 2 provided 35 alerts, with 34 of them identified as false, resulting in a FAR of 97% under rainy conditions versus 40% under dry conditions. When exposure was considered, System 2 produced approximately 12.7 stopped-vehicle alerts and 12.3 false stopped-vehicle alerts per 100 rainy camera-hours, compared with approximately 3.9 alerts and 1.6 false alerts per 100 dry camera-hours.

### 4.5. Statistical Model of Contributing Factors to FAR

A binary logit model was developed to examine the factors contributing to false alerts in stopped vehicle detections. Table 5 shows the estimated coefficients, standard errors, z-statistics, *p*-values, odds ratios (ORs), and 95% confidence intervals (95% CIs) of the estimated model that uses all data after excluding detections from the 0389-CCTV camera across all three systems to provide a consistent comparison. The dependent (predicted) variable of the model is whether an alert is false or not (1 = False Alert, 0 = True Alert). The odds ratio (OR) quantifies how a one-unit increase in an independent variable affects the odds of a false alert (odds = the likelihood of a false alert relative to a true alert) and OR = 1 represents no effect, OR > 1 represents the factor increases the risk of a false alert, and OR < 1 represents the factor decreases the risk of a false alert.

The modeling results show the following findings:

- Utilized System: The coefficients and odds ratios indicate that System 2 had 3.85 times the odds of generating a false alert compared with System 1 (95% CI: 1.69–8.76; *p* = 0.001). System 3 had 9.07 times the odds of generating a false alert compared with System 1 (95% CI: 3.12–26.34; *p* < 0.001).
- Study Sites: The model indicates that different cameras exhibit varying likelihoods of generating false alerts. These differences may be attributed to site-specific traffic patterns, roadway geometry, and/or camera viewing angles.
- Detection Location: Alerts associated with objects on a travel lane had substantially higher odds of being false than alerts associated with other locations, such as shoulders (OR = 54.56; 95% CI: 22.62–131.62; *p* < 0.001). This result indicates that detections occurring on travel lanes are more likely to result in false alerts compared to those in other areas, such as the shoulder. This finding may be attributed to the frequent stop-and-go traffic patterns observed on travel lanes.
- Rain: Rainy conditions were associated with higher odds of a false alert than dry conditions (OR = 83.53; 95% CI: 10.33–675.79; *p* < 0.001). However, only 36 alerts occurred during rainy conditions, of which 35 were false. This small and highly imbalanced rainy-condition sample produced a wide confidence interval and indicates that the estimated effect is imprecise. Therefore, although the direction of the association is clear in the study data, its magnitude should be interpreted cautiously.
- Peak Hours: Alerts generated during peak hours had 2.45 times the odds of being false compared with alerts generated during off-peak periods (95% CI: 1.27–4.70; *p* = 0.007), after controlling for the other variables. This finding may be associated with increased congestion and stop-and-go traffic during peak periods.
- Night: No significant impact of night conditions on false detections was found, as the corresponding model coefficient was not statistically significant.

### 4.6. Incident Matching Rate

A sensitivity analysis was conducted using all true stopped vehicle alerts to assess the sensitivity of the matching rate to the selected spatial and temporal thresholds. The study attempted to match the incidents detected using computer-vision-based AID systems with incident records archived in the FDOT database based on two incident information sources (FDOT Recorded and Waze Provided). Table 6 shows the matching rates obtained under different combinations of spatial and temporal thresholds. The results show that the matching rate increased as the spatial and temporal matching thresholds were increased as expected. Under a selected criterion of 0.5 mile and ±30 min, the matching rates were 20.6%, 17.8%, and 29.6% for Systems 1, 2, and 3, respectively. When the criteria were tightened to 0.25 mile and ±15 min, the corresponding matching rates decreased to 8.4%, 8.3%, and 18.5%. In contrast, when the criteria were relaxed to 1.0 mile and ±60 min, the matching rates increased to 44.9%, 43.3%, and 51.9%, respectively. These results indicate that the matching rate is sensitive to the selected spatial and temporal matching windows. The spatial and temporal thresholds of 0.5 miles and ±30 min were selected for the rest of the analysis based on discussion with traffic management center (TMC) operators because they reflect the expected uncertainty in incident location and reporting time.

**Figure 4:**
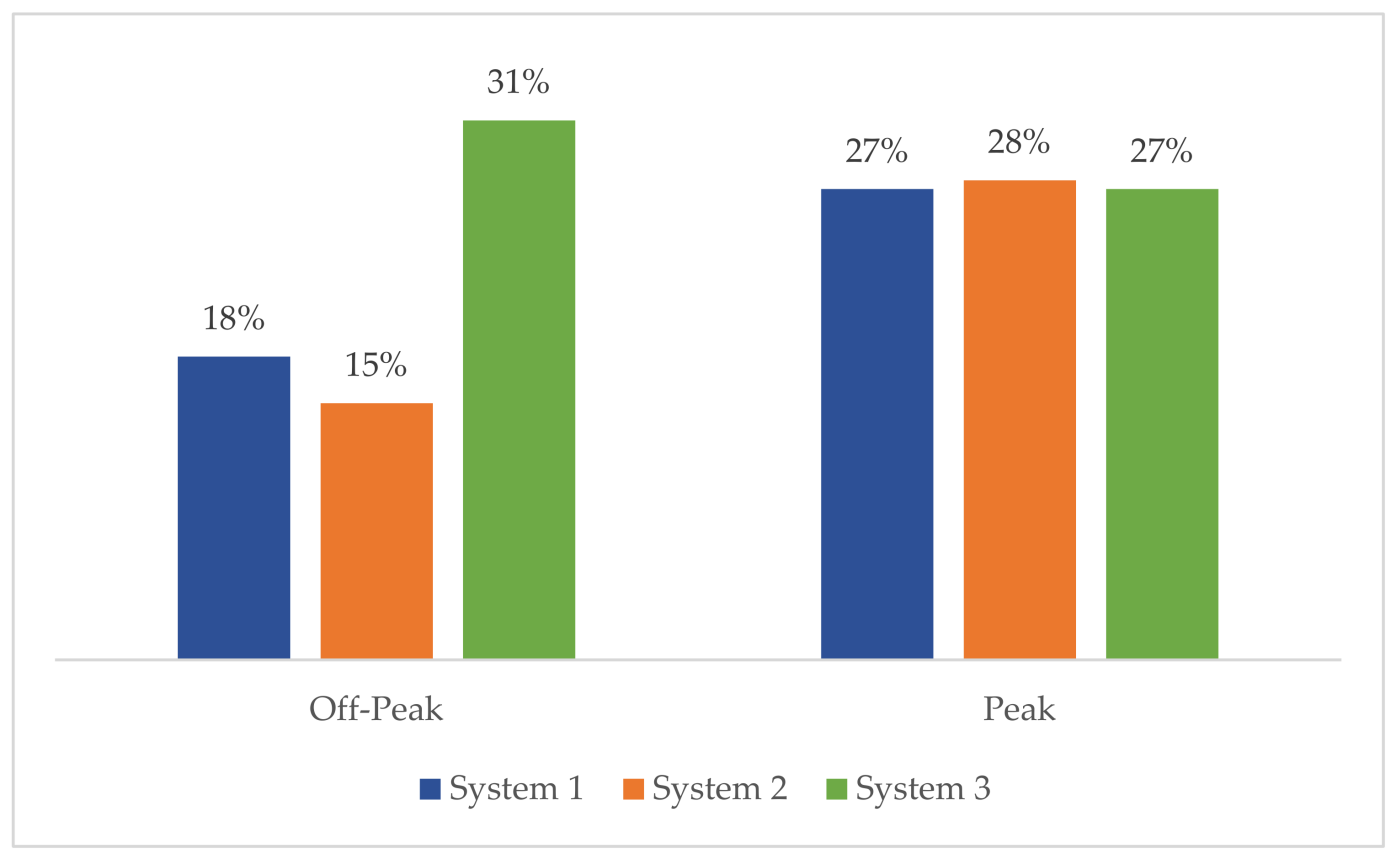
Matching rate for stopped vehicle incidents.

Using the selected 0.5-mile and ±30-min matching criterion, the next step was to calculate the matching rate by comparing the true alerts from each system with the incident records in the FDOT database. This comparison focused on AID of stopped vehicles and aimed at determining the additional detection capabilities provided by the AID systems. Figure 4 indicates that the off-peak matching rates were 18%, 15%, and 31% for Systems 1, 2, and 3, respectively. During the peak periods, the matching rates were 27%, 28%, and 27% for the three systems, respectively.

**Figure 5:**
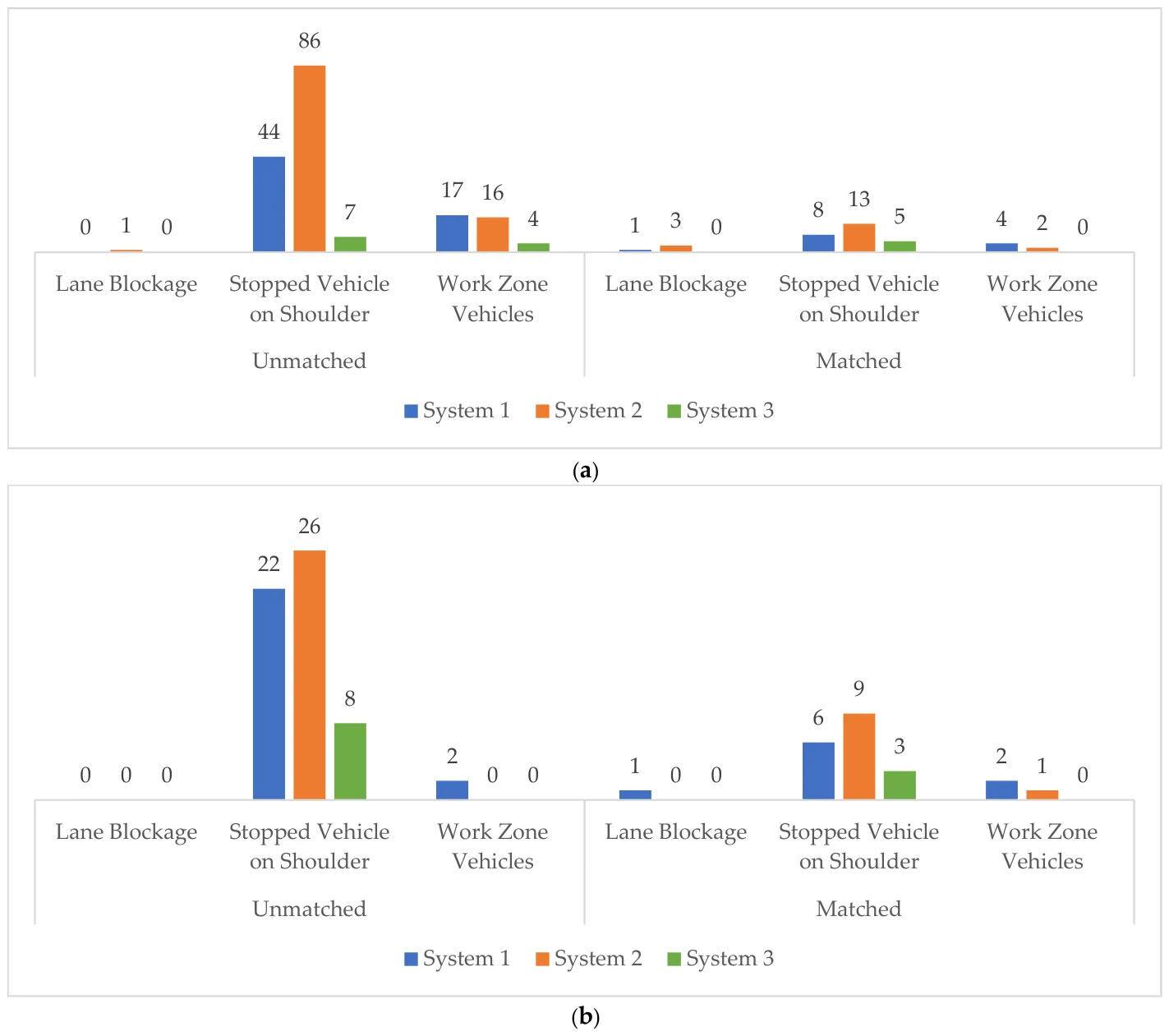
Matching context for stopped vehicle incidents. (**a**) Off-Peak Hours. (**b**) Peak Hours.

As shown in Figure 5a, the unmatched alerts are mainly associated with stopped vehicles on the shoulder and work zone vehicles. A large proportion of unmatched incidents occurred during the off-peak period. Some of the unmatched incidents were possibly minor incidents that do not meet the severity or duration thresholds required for logging them by the TMC operator in the management software. These could include for example disabled vehicles temporarily stopped on the shoulder. Since the length of the video recordings is only 30 s after incident detection, it was not possible to verify the durations of the unmatched events. The high number of unmatched events for the off-peak period that includes the nighttime may indicate a lower level of TMC operations in this period, pointing to potential benefits of AID at off-peak.

### 4.7. Latency of Detection

The latency of detection was assessed by comparing the time difference between the AID of true alerts and the corresponding matched incidents in the FDOT database. A negative difference indicates that the evaluated system detected the incident earlier than the incident time reported in the existing database (Waze-Provided or FDOT-Recorded). It should be mentioned here that most of the matched incidents are shoulder incidents and may be minor incidents with lower priority, which may increase the time from their occurrence until their recording in the FDOT central software.

Table 7 summarizes the descriptive statistics of AID latencies compared to the timestamps in the existing database. Due to the presence of extreme values, the median rather than the mean was used in comparison because the median is less affected by extreme values. The differences in the detection latency median between the AID system recording and Waze-Provided timestamps were small ranging from −56 s to 6 s, depending on the system. However, the differences in the detection latency median between the AID-detected and the FDOT-Recorded timestamps were much higher ranging from −713 s to 329 s. One-tailed hypothesis testing was used to determine if there is a significant difference in the median. Because the sample size was small and the distribution was skewed, this study used the one-tailed permutation test, a non-parametric method. The hypothesis states that:

**H0.**  *No difference between AID time and existing reporting time.*

**Ha.**  *AID time is earlier than the existing reporting time.*

The testing results in Table 6 show that no statistically significant evidence of earlier detection was found for the three systems compared to FDOT detection and Waze detection records. These nonsignificant results should not necessarily be interpreted as evidence of equivalent detection times because the statistical power was limited by the small samples. This limitation is particularly important for System 3, for which only six incidents were matched to FDOT and seven were matched to Waze. With such small samples, the permutation distribution is discrete and highly sensitive to individual observations, making the resulting *p*-values and median estimates unstable. System 3 results should therefore be considered descriptive rather than conclusive.

## 5. Discussion and Conclusions

The three tested systems were able to use video streams from six PTZ CCTV cameras for incident detection when these cameras were on predefined presets. To reduce false alerts, there should be a parameter setting in the AID systems to detect when the cameras are not on preset and stop issuing alerts then. While some (but not all) of the tested systems were reported to have this capability, this functionality was not tested during the pilot study.

The AID systems had limited detection zones, ranging from 200 to 700 feet in length in the field of view of the camera preset. The average distance between two CCTV cameras in urban areas is approximately one mile and can be significantly longer in rural areas. As a result, it is challenging to achieve full corridor coverage with the AID systems but they could be used to cover selected high-incident locations.

This study evaluated AID systems to detect stopped vehicles and pedestrian presence. The frequency of alerts and false alarms is a function of vehicle stoppage time thresholds. Only one of the three systems allows the user to change these thresholds. It is recommended that all systems provide this capability to optimize system performance.

The AID system accuracy varies by study site, likely due to traffic patterns, roadway geometry, and camera angles. Two major factors contribute to AID false alerts: stop-and-go traffic and other object presence including light reflection and glare.

The three tested systems performed differently. System 2 had the highest number of true stopped vehicle incident detections, generating more detections than the other two systems but also a high number of alerts, particularly with one of the camera locations in the off-peak period. System 1 produced the second highest number of true alerts and the lowest number of false alerts. System 3 produced the lowest number of alerts and had a relatively high FAR in the peak period. In terms of detecting pedestrian presence, System 3 demonstrated the best performance across different scenarios with zero false alerts for this type of detection, whereas Systems 1 and 2 generated some pedestrian false alerts.

It appears that the impact of night conditions varies depending on the system with System 3 seemingly experiencing a higher reduction in alerts of stopped vehicles and System 2 having the highest impact in terms of FAR due to detecting other objects such as light reflections and glares as incidents. Rainy conditions also increased the false alerts for System 2. Due to the small sample size, it may be difficult to draw a conclusion about the impact of weather on AID.

The three systems were able to detect additional incidents not reported in the databases of the existing incident management system of the TMC. These incidents appear to be mainly shoulder incidents and work zones. Some of these incidents could be considered as minor incidents such as disabled vehicles.

## Figures and Tables

**Table 1:** Summary of AID systems.

Feature	System 1	System 2	System 3
Detectable Incident Types	Crash, wrong way, stopped/slow vehicles, debris, queue	Crash, wrong way, stopped/slow vehicles, debris, queue	Crash, wrong-way, stopped vehicles, queue
Implementable Facility Types	Interstates, arterials, intersections, bridges, at-grade crossings	Interstates, arterials, intersections, bridges, at-grade crossings	Interstates, intersections
Environmental Conditions	Dry and rainy conditions	Dry and rainy conditions utilize headlight tracking at night	Dry and rainy conditions utilize headlight tracking at night
Traffic Conditions	Free-flow and “stop-n-go”	Free-flow and “stop-n-go”	Free-flow and “stop-n-go”
PTZ Camera Compatibility	Works with PTZ, provides auto-calibration	Prefers fixed cameras, partially works with PTZ	Prefers fixed cameras, partially works with PTZ
User Interface for	Webpage	Windows Client	Webpage
Detection Alerts	Email/Text Message	Email	Email/Text Message
Computing Needs	CPU	CPU	GPU
Inputs	Video stream	Video stream	Video stream
Calibration Time	2–4 weeks	2–4 weeks	2–4 weeks

**Table 2:** Number of detected incidents.

VID System	Observation Days	Total No. of Incidents		On-Preset Detected Incidents	
		Stopped Vehicle	Pedestrian	Stopped Vehicle	Pedestrian
System 1	46	352	22	163 (3.5 per day)	18 (0.4 per day)
System 2	46	978	127	341 (7.4 per day)	32 (0.7 per day)
System 3	43 ^1^	308	105	84 (2.0 per day)	29 (0.7 per day)

^1^ Excluding days and data for 15 to 24 February 2025 during which System 3 was upgraded and 27 February to 5 March 2025 during the backup process.

**Table 3:** Verified causes of false detections of stopped vehicles.

Period	Verified Cause	System 1 (Excluding 0389—CCTV)		System 2 (Excluding 0389—CCTV)		System 3 (Excluding 0389—CCTV)	
		Obs. ^1^	Rate ^2^	Obs. ^1^	Rate ^2^	Obs. ^1^	Rate ^2^
Off-peak	Other objects	4	17%	58	74%	8	73%
	Stop-n-go	19	83%	20	26%	3	27%
peak	Other objects	4	29%	21	55%	5	17%
	Stop-n-go	10	71%	17	45%	25	83%

^1^ Obs. = Number of False Detections. ^2^ Rate = number of false detections associated with a verified context divided by the total number of false detections.

**Table 4:** FAR by environmental conditions.

Incident Type	Condition	System 1 (Excluding 0389—CCTV)	System 2 (Excluding 0389—CCTV)	System 3 (Excluding 0389—CCTV)
Pedestrian	Day	2/8 = 25%	4/20 = 20%	0/21 = 0%
	Night	2/4 = 50%	0/1 = 0%	0/5 = 0%
	Dry	4/12 = 33%	4/21 = 19%	0/26 = 0%
	Rain	NA	NA	NA
Stopped vehicle	Day	24/75 = 32%	52/126 = 41%	36/50 = 72%
	Night	13/37 = 35%	64/116 = 55%	5/10 = 50%
	Dry	36/111 = 32%	82/207 = 40%	41/60 = 68%
	Rain	1/1 = 100%	34/35 = 97%	NA

NA = No detections occurred.

**Table 5:** Estimated Binary Logit Model for Stopped Vehicle False Alert.

Variable	Coefficient	Odds Ratio	95% CI for OR	Standard Error	z-Statistic	*p*-Value
System 1	Baseline					
System 2	1.35	3.85	1.69–8.76	0.42	3.21	0.001
System 3	2.20	9.07	3.12–26.34	0.54	4.05	<0.001
0286-CCTV	Baseline					
0225-CCTV	−0.23	0.79	0.31–2.00	0.47	−0.50	0.620
0306-CCTV	−1.97	0.14	0.02–0.87	0.93	−2.11	0.035
0376-CCTV	0.90	2.47	1.01–6.05	0.46	1.98	0.048
0440-CCTV	1.43	4.17	1.85–9.38	0.41	3.45	<0.001
On travel lane	4.00	54.56	22.62–131.62	0.45	8.90	<0.001
Rain	4.43	83.53	10.33–675.79	1.07	4.15	<0.001
Peak hours	0.89	2.45	1.27–4.70	0.33	2.68	0.007
Night	0.08	1.08	0.56–2.08	0.33	0.23	0.815
Constant	−3.34	0.04	0.01–0.10	0.54	−6.18	<0.001

**Table 6:** Sensitivity of Matching Rates to Spatial and Temporal Thresholds.

Matching Criteria	System 1	System 2	System 3
0.25 mi, ±15 min	8.4%	8.3%	18.5%
0.25 mi, ±30 min	16.8%	13.4%	29.6%
0.25 mi, ±45 min	20.6%	15.9%	33.3%
0.25 mi, ±60 min	27.1%	21.0%	37.0%
0.50 mi, ±15 min	12.1%	10.8%	22.2%
0.50 mi, ±30 min	20.6%	17.8%	29.6%
0.50 mi, ±45 min	28.0%	25.5%	33.3%
0.50 mi, ±60 min	35.5%	31.8%	44.4%
0.75 mi, ±15 min	14.0%	14.0%	25.9%
0.75 mi, ±30 min	26.2%	22.9%	33.3%
0.75 mi, ±45 min	33.6%	29.3%	37.0%
0.75 mi, ±60 min	41.1%	37.6%	51.9%
1.00 mi, ±15 min	15.9%	16.6%	25.9%
1.00 mi, ±30 min	29.0%	27.4%	33.3%
1.00 mi, ±45 min	36.4%	33.8%	37.0%
1.00 mi, ±60 min	44.9%	43.3%	51.9%

**Table 7:** Statistics of AID latency and hypothesis test.

VID System	Item		FDOT	WAZE
System 1	Statistics	Sample Size	14	16
		Min/Max (s)	−1659/1739	−1599/1411
		1st Quartile	−349.5	−565.5
		3rd Quartile	1316.50	559.8
		Mean (s)	332.4	−58.5
		Std. Deviation (s)	1129.80	932.9
		Median difference (s)	329	−56
	One-tailed Permutation Test	*p*-value	0.83	0.45
		Significant?	No	No
System 2	Statistics	Sample Size	19	21
		Min/Max (s)	−1707/1783	−1703/1483
		1st Quartile	−948.5	−679
		3rd Quartile	605.5	636
		Mean (s)	−169.5	−22
		Std. Deviation (s)	1147.50	900.4
		Median difference (s)	−713	6
	One-tailed Permutation Test	*p*-value	0.15	0.68
		Significant?	No	No
System 3	Statistics	Sample Size	6	7
		Min/Max (s)	−1628/605	−1531/845
		1st Quartile	−1020.30	−636.5
		3rd Quartile	177.8	327.0
		Mean (s)	−492.5	−194.3
		Std. Deviation (s)	870.6	810.9
		Median difference (s)	−606.5	−55
	One-tailed Permutation Test	*p*-value	0.094	0.50
		Significant?	No	No

## Data Availability

The original contributions presented in this study are included in the article. Further inquiries can be directed to the corresponding author.
